# Safety and efficacy of Pazopanib in advanced soft tissue sarcoma: PALETTE (EORTC 62072) subgroup analyses

**DOI:** 10.1186/s12885-019-5988-3

**Published:** 2019-08-13

**Authors:** Axel Le Cesne, Sebastian Bauer, George D. Demetri, Guangyang Han, Luca Dezzani, Qasim Ahmad, Jean-Yves Blay, Ian Judson, Patrick Schöffski, Massimo Aglietta, Peter Hohenberger, Hans Gelderblom

**Affiliations:** 1 Département d’Oncologie Médicale, Gustave Roussy, 114 Rue Edouard Vaillant, 94805 Villejuif Cedex, Villejuif, France; 2 Sarcoma Center, West German Cancer Center, University of Duisburg-Essen, Hufelandstraße 55, 45147 Essen, Germany; 3 Ludwig Center at Harvard, Harvard Medical School and Department of Medical Oncology, Dana-Farber Cancer Institute, 450 Brookline Avenue, Boston, MA 02215 USA; 4 Novartis Oncology, One Health Plaza, East Hanover, NJ 07936 USA; 5 Department of Medical Oncology, Leon Berard Center, 28, rue Laennec 2 69373 Lyon Cedex 08, Lyon, France; 6 The Institute of Cancer Research, Royal Marsden NHS Foundation Trust, 123 Old Brompton Road, London, SW7 3RP UK; 7 Department of General Medical Oncology, University Hospitals Leuven, Leuven Cancer Institute, Herestraat 49, B-3000 Leuven, Belgium; 8 Department of Oncology, University of Torino and Candiolo Cancer Center FPO-IRCCS, 10060 Candiolo, (Torino) Italy; 9 Division of Surgical Oncology & Thoracic Surgery, Mannheim University Medical Center, Theodor Kutzer Ufer 1, D-68165 Mannheim, Germany; 10 Department of Medical Oncology, Leiden University Medical Center, Albinusdreef 2, 2333 Leiden, ZA Netherlands

**Keywords:** Pazopanib, Advanced soft tissue sarcoma, Progression-free survival, PALETTE subgroup analysis

## Abstract

**Background:**

PALETTE is a phase 3 trial that demonstrated single-agent activity of pazopanib in advanced soft tissue sarcomas (aSTS). We performed retrospective subgroup analyses to explore potential relationships between patient characteristics, prior lines of therapy, dose intensity, and dose modifications on safety and efficacy of pazopanib in aSTS.

**Methods:**

PALETTE compared pazopanib with placebo in patients with aSTS (age ≥ 18 years) whose disease had progressed during or following prior chemotherapy. In these subgroup analyses, median progression-free survival (mPFS) among patients receiving pazopanib was the efficacy outcome of interest. Adverse events (AEs) were also compared within subgroups. All analyses were descriptive and exploratory.

**Results:**

A total of 246 patients received pazopanib in the PALETTE study. The mPFS was longer in patients who had only 1 prior line versus 2+ prior lines of therapy (24.7 vs 18.9 weeks, respectively); AE rates were similar regardless of number of prior lines of therapy. The mPFS was similar in patients aged < 65 and ≥ 65 y (20.0 and 20.1 weeks, respectively). Although AEs leading to study discontinuation were higher in older patients (≥65 y, 30%; < 65 y, 17%), rates of dose reductions, dose interruptions, and serious AEs were similar between the 2 age groups. No reduction in mPFS was noted in patients requiring dose reductions or dose interruptions to manage toxicities.

**Conclusions:**

Longer mPFS was observed in patients receiving pazopanib following only 1 line of therapy. Additionally, mPFS with pazopanib was maintained regardless of patient age or dose modifications used to manage toxicity.

**Trial registration:**

NCT00753688, first posted September 16, 2008 (registered prospectively).

**Electronic supplementary material:**

The online version of this article (10.1186/s12885-019-5988-3) contains supplementary material, which is available to authorized users.

## Background

Pazopanib is an oral, small-molecule tyrosine kinase inhibitor (TKI) targeting vascular endothelial growth factor (VEGF) receptors (VEGFR-1, − 2, and − 3), platelet-derived growth factor (PDGF) receptors (PDGFR-alpha and -beta), fibroblast growth factor receptor, and KIT [1]. The predominant role of VEGF and PDGF in tumor angiogenesis and their expression across many soft tissue sarcoma (STS) subtypes provided a strong rationale for the evaluation of pazopanib in STS. In a placebo-controlled, randomized, phase 3 trial in patients with advanced STS (aSTS; excluding liposarcomas and gastrointestinal stromal tumor [GIST]), pazopanib administration led to significantly improved progression-free survival (PFS) compared with placebo [2]. These results led to the US regulatory approval of pazopanib for treating patients with aSTS who have previously received chemotherapy [3]. The EMA has approved pazopanib for adults with selected subtypes of aSTS following prior chemotherapy for metastatic disease or after progression within 12 months after (neo)adjuvant therapy [4]. Pazopanib was the first molecularly targeted agent approved for treating aSTS at a time when treatment options following failure of first-line chemotherapy (the most common first-line treatment) were very limited.

The incidence of STS increases with age, with approximately 50% of newly diagnosed patients being older than 65 years [5]. In addition to the high-grade and/or high-stage STS presentation in elderly patients versus younger patients [6, 7], poor prognosis in older patients might also relate to undertreatment based on misconceptions of tolerability and safety [8]. Older patients are less likely to be treated with adjuvant chemotherapy, radiotherapy, or definitive surgery [9]. Also, comorbidities are generally more common in older rather than younger patients. Underrepresentation of elderly patients further compromises the generalizability of the clinical trial findings to clinical practice [10]. Number of prior lines of therapy also influences STS outcomes. In a retrospective study evaluating novel targeted therapies in patients with aSTS after progression from US FDA-approved therapies, patients who had received 2 or fewer prior lines of treatment had substantially improved overall survival (OS) in comparison with patients who had received 3 or more prior lines of treatment [11].

Flexibility of pazopanib dosing in patients with aSTS may be crucial for optimal treatment and tolerability in this setting. In the PALETTE trial, dose interruptions and dose reductions were allowed to manage adverse events (AEs). Pazopanib treatment was temporarily interrupted in 49% of patients, and 39% of patients received dose reductions. A definitive treatment discontinuation due to AEs related to pazopanib occurred in 14% of patients [2]. However, limited data have been published to date on pazopanib efficacy and safety in patients undergoing dose interruptions and dose reductions.

Using data from the PALETTE trial, we investigated pazopanib efficacy and safety in specific subgroups of patients with aSTS. Understanding the influence of age, prior lines of therapy, dosing intensity, and dose modifications on pazopanib outcomes could potentially alleviate safety and tolerability concerns and guide optimal usage of pazopanib in patients with aSTS.

## Methods

### Study design

PALETTE (EORTC 62072) was a randomized, double-blind, placebo-controlled, phase 3 trial, conducted by the Soft Tissue and Bone Sarcoma Group of the European Organization for Research and Treatment of Cancer between October 2008 and November 2010. Patients were randomized 2:1 to receive either pazopanib 800 mg once daily or placebo, with no subsequent cross-over. As part of the original study [2], all patients provided written informed consent and the trial was approved by all relevant review bodies. Because the subgroup analyses used existing data from the primary study, additional consent was not required. The study was conducted in accordance with the Declaration of Helsinki and Good Clinical Practice guidelines. Full details of the PALETTE study design, including inclusion and exclusion criteria, have been published previously [2].

Subgroups of interest in our current post hoc analyses were based on the following metrics: prior lines of therapy (only 1 prior line of therapy vs 2+ prior lines of therapy), age (< 65 years vs ≥65 years) dose intensity (dose < 400 mg, ≥400 mg to < 600 mg, and ≥ 600 mg to ≤800 mg), and dose modifications (no dose reduction vs dose reductions; no dose interruption vs dose interruptions) among patients randomized to receive pazopanib in the PALETTE trial. All subgroup analyses were exploratory and descriptive in nature, with no statistical hypothesis testing.

### Eligibility criteria

Key inclusion criteria included patients of age ≥ 18 years with aSTS and disease progression within 6 months prior to receiving study drug or within 12 months of previous adjuvant treatment, ≥1 regimen containing anthracycline, and ≤ 4 lines of prior systemic therapy for metastatic disease. No more than 2 previous lines should have been combination regimens, and (neo)adjuvant/maintenance treatments were not counted toward this criterion. Key exclusion criteria included patients with adipocytic sarcoma, embryonal rhabdomyosarcoma, chondrosarcoma, osteosarcoma, and GIST. Patients with clinically abnormal cardiac function or poorly controlled hypertension were also excluded. Patients who had had a cerebrovascular accident, pulmonary embolism, untreated deep venous thrombosis, or clinically significant gastrointestinal disorders in the past 6 months were ineligible.

### Criteria for dose modifications

Dose interruptions or reductions were permitted following potential drug-related toxicities including but not limited to hypertension, proteinuria, hepatotoxicity, bleeding events, thrombosis, and thrombocytopenia/neutropenia. In cases wherein a dose reduction was necessary, 2 stepwise dose reductions were permitted: initially to 600 mg and subsequently to 400 mg. If the toxicity did not recur or worsen, the doses could be increased stepwise back to 600 mg and 800 mg after monitoring for 10–14 days at each step. If a patient’s treatment had been interrupted > 14 days due to toxicity, resumption of treatment was based on patient’s condition and recovery from toxicity at reduced dose. An additional table provides a detailed description of protocol-defined and prespecified dose modifications for potential treatment-related AEs (see Additional file 1).

### Study endpoints

The primary objective of the PALETTE trial was to demonstrate superiority in PFS of pazopanib over placebo. In this subgroup analysis, the efficacy outcome of interest was median PFS (mPFS) among pazopanib recipients in the PALETTE trial. Adverse events were also compared within the subgroups.

### Statistical analysis

Efficacy was evaluated in the intent-to-treat population, which included all patients who were randomized to treatment. Although the PALETTE study was stratified according to number of previous lines of systemic therapy and was powered to detect a 15% difference in PFS (pazopanib versus placebo arms) at 6 months, the study was not powered for any subgroup analyses. The safety population, which was defined as all patients who were administered their allocated treatment and had received at least 1 dose of the study drug, was used for all safety analyses.

## Results

### Patient characteristics

A total of 246 patients were randomized to the pazopanib arm and represented the intention-to-treat (ITT) population. At baseline, the mean age was 54 (±15) years, 60% of patients were female, and 25% of the patients were ≥ 65 years of age (Table 1). At the time of the primary analysis, median follow-up was 14.9 months (interquartile range, 11.0–18.2) in the pazopanib group; disease progression was documented in 168 recipients and 137 patients had died.

**Table 1:** Baseline Characteristics of Pazopanib Recipients in the PALETTE Trial (ITT Population)

	Pazopanib Arm (*n* = 246)
Age, y, mean (SD)	54.0 (14.9)
< 65 y, n (%)	184 (75)
≥65 y, n (%)	62 (25)
Female, n (%)	147 (60)
Weight, kg, mean (SD)	71.5 (16.9)
STS subtypes, n (%)	
Leiomyosarcoma	109 (44)
Synovial sarcoma	25 (10)
Other STS histologies	112 (46)
Time since initial diagnosis, mo, median (IQR)	26.6 (14.5–46.1)
Time since last progression, mo, median (IQR)	0.7 (0.3–1.1)
Prior lines of therapy, n (%)	
1	110 (45)
2 or more	136 (55)

*SD* standard deviation, *STS* soft tissue sarcoma, *IQR* interquartile range

### Subgroup analysis by age

Among patients receiving pazopanib, 184 patients were < 65 years of age and 62 patients were ≥ 65 years of age. The mPFS was similar in the 2 age subgroups (age < 65 years, 20.0 [95% CI, 17.9–22.0] weeks and age ≥ 65 years, 20.1 [95% CI, 11.7–31.6] weeks, respectively). Treatment-related AEs occurred in 93% of patients < 65 years of age versus 85% of patients ≥65 years of age (Table 2). The AEs leading to study discontinuation occurred at a higher rate in older (≥65 years) versus younger (< 65 years) patients (30% vs 17%, respectively). However, rates of dose reductions, dose interruptions, and serious AEs leading to study discontinuation were similar between the 2 age groups (Table 2).

**Table 2:** Adverse Events in Pazopanib Recipients by Age Subgroups (Safety Population)

Events, n (%)	Age < 65 Years (*n* = 180)	Age ≥ 65 Years (*n* = 60)
Any on-therapy AE	178 (99)	59 (98)
AEs related to study treatment	168 (93)	51 (85)
AEs leading to permanent discontinuation or early withdrawal	30 (17)	18 (30)
AEs leading to dose reduction	60 (33)	17 (28)
AEs leading to dose interruption/delay	89 (49)	31 (52)
Any SAE	75 (42)	24 (40)
SAEs related to study treatment	40 (22)	17 (28)
Fatal SAEs	7 (4)	1 (2)
Fatal SAEs related to study treatment	1 (< 1)	0 (0)

*AE* adverse event, *SAE* serious adverse event.

### Subgroup analysis by prior lines of therapy

Among patients receiving pazopanib, 110 patients had received 1 prior line of therapy and 136 patients had received 2 or more prior lines of therapy. The mPFS was higher in patients with 1 prior line of therapy (24.7 weeks [95% CI, 19.6–27.4]) versus patients with 2 or more prior lines of therapy (18.9 weeks [95% CI, 11.9–20.1]). Rates of AEs were similar between the 2 subgroups (Table 3).

**Table 3:** Adverse Events in Pazopanib Recipients by Number of Prior Lines of Therapy (Safety Population)

Events, n (%)	1 prior line (*n* = 109)	2+ prior lines (*n* = 131)
Any on-therapy AE	108 (> 99)	129 (98)
AEs related to study treatment	101 (93)	118 (90)
AEs leading to permanent discontinuation or early withdrawal	24 (22)	24 (18)
AEs leading to dose reduction	38 (35)	39 (30)
AEs leading to dose interruption/delay	59 (54)	61 (47)
Any SAE	47 (43)	52 (40)
SAEs related to study treatment	26 (24)	31 (24)
Fatal SAEs	5 (5)	3 (2)
Fatal SAEs related to study treatment	0 (0)	1 (< 1)

*AE* adverse event, *SAE* serious adverse event.

### Subgroup analysis by dose intensity

Among patients receiving pazopanib, the majority (*n* = 234) received doses between 600 and 800 mg daily. Only 4 patients received doses between 400 and 600 mg daily, and 7 patients received doses less than 400 mg daily. Among patients receiving the highest doses, mPFS was 20.1 weeks (95% CI, 17.9–21.3). Patients who received between 400 and 600 mg pazopanib daily had mPFS of 25.3 weeks (95% CI, 8.1–38.1), and mPFS was 5.1 weeks (95% CI, 3.3–7.0) in patients who received less than 400 mg pazopanib daily.

### Subgroup analysis by dose modifications

Among patients receiving pazopanib, patients with 1 or more dose reductions had substantially higher mPFS than patients with no dose reductions (27.7 weeks [95% CI, 21.1–35.7] vs 11.9 weeks [95% CI, 8.9–19.3], respectively) (Table 4). Similarly, patients with 1 or more dose interruptions had substantially higher mPFS than patients with no dose interruptions (21.3 weeks [95% CI, 20.1–27.7] vs 11.0 weeks [95% CI, 8.1–19.3], respectively) (Table 4).

**Table 4:** Progression-Free Survival in Pazopanib Recipients by Dose Modifications (ITT Population)

Dose Modifications	n (%)	mPFS, weeks (95% CI)
Dose reductions		
No dose reduction	154 (100)	11.9 (8.9–19.3)
Progressed or Died	104 (68)	
≥ 1 dose reductions	92 (100)	27.7 (21.1–35.7)
Progressed or Died	59 (64)	
1 dose reduction	54 (100)	27.1 (20.1–35.7)
Progressed or Died	29 (54)	
2 or more dose reductions	38 (100)	28.1 (20.1–38.1)
Progressed or Died	30 (79)	
Dose interruptions		
No dose interruption	107 (100)	11.0 (8.1–19.3)
Progressed or Died	71 (66)	
≥ 1 dose interruptions	139 (100)	21.3 (20.1–27.7)
Progressed or Died	92 (66)	
1 dose interruption	69 (100)	20.0 (12.4–21.3)
Progressed or Died	45 (65)	
2 or more dose interruptions	70 (100)	27.7 (21.0–35.6)
Progressed or Died	47 (67)	

*CI* confidence interval, *ITT* intention to treat

## Discussion

In this PALETTE subgroup analysis, the magnitude of clinical benefit observed with pazopanib was similar between elderly and younger patients. Increasing age was apparently unrelated to mPFS. Higher rates of AEs leading to study discontinuation in older patients are not surprising, likely due to a higher prevalence of comorbidities and reduced tolerability in elderly compared with younger patients. However, rates of dose reductions, dose interruptions, and serious AEs were similar between the two age groups. Retrospective analyses of patients receiving other therapies for aSTS have reported similar findings of a higher rate of AEs in elderly patients compared with their younger counterparts [12, 13]. Because the AE profile of each agent may vary, the individual safety profile of each agent should inform treatment decisions in the elderly, especially in the presence of comorbidities.

As might be expected, the number of lines of previous systemic therapy was a significant prognostic factor for PFS among pazopanib recipients in the PALETTE study (0–1 vs 2–4 prior lines of therapy, HR [95% CI]: 0.72 [0.53–0.99]; *P* = 0.04) [2]. This finding is in agreement with “real-world evidence” demonstrating that patients with aSTS exhibit a decline in mPFS with each additional line of previous therapy [14]. The randomized, phase 2 EPAZ trial (NCT01861951) demonstrated that pazopanib was noninferior to doxorubicin with respect to PFS in the first-line treatment of STS in patients more than 60 years of age [15]. Recent studies have suggested that in some cases systemic anticancer therapy may promote progression of cancer rather than only influencing cancer evolution [16–18]. STS are characterized by tumor heterogeneity, and evolution of tumor heterogeneity in response to therapy is a well-established phenomenon [19]. Mutagenesis driven by cytotoxic therapies or by acquired chromosomal instability could drive clonal selection, leading to greater intratumoral heterogeneity and thereby increasing the likelihood of resistance to subsequent treatment [19]. Treatment sequence had no effect on pazopanib’s safety profile, as evidenced by similar AE rates between the 2 subgroups based on prior lines of therapy.

In a subgroup analysis by pazopanib dose, patients receiving a daily dose between 400 and 600 mg had higher mPFS than patients receiving a daily dose between 600 and 800 mg or a daily dose of less than 400 mg; however, due to the small numbers of patients in the lower-dose subgroups, these results should be interpreted cautiously. Patients with metastatic renal cell carcinoma (RCC) receiving a lower starting dose of first-line pazopanib fare worse compared with those receiving a standard dose [20]. At a median follow-up of 13.9 months, patients receiving reduced starting dose (400 or 600 mg/day) versus standard dose (800 mg/day) have shown substantially reduced objective response rates (19% vs 44%, respectively) and increased discontinuation rates due to progressive disease (44% vs 28%, respectively) [20].

Intriguingly, patients receiving pazopanib with 1 or more dose reductions or dose interruptions because of drug toxicity had improved mPFS compared with patients in whom dose modifications were not required. These findings are consistent with those of the COMPARZ study in advanced renal cell carcinoma, wherein longer mPFS in pazopanib-treated patients was observed when dose modification was required because of toxicity, suggesting that patients are not at a disadvantage when such dose reductions or interruptions are needed [21]. In this context, “on-target” toxicities of TKIs have been suggested as potential indicators of efficacy [22]. In a pharmacokinetics/pharmacodynamics study in patients with RCC, the threshold concentration for pazopanib efficacy overlapped with concentrations at which toxicity occurs [23]. Evidence from previous reports and this study suggests that pazopanib recipients with no toxicity signs and symptoms (and thus not requiring a dose modification) may have suboptimal therapeutic drug exposure. However, the post hoc observational analysis in the current study did not account for the timing of dose modifications on treatment efficacy. A potential for bias due to early discontinuation in the groups with no dose reductions or interruptions subgroups cannot be ruled out, and hence, the effects of dose reductions and dose interruptions on mPFS outcomes need cautious interpretation.

This study has some additional limitations. Only data from subgroups of patients that received pazopanib were evaluated. All analyses were post hoc, descriptive, and exploratory in nature, and were not designed to permit statistical comparison across subgroups. For this reason, the possibility of bias in the descriptions of treatment effects in the post-randomization subgroups cannot be ruled out. Our findings therefore should be considered preliminary and will need to be confirmed in “real-world” settings.

## Conclusions

In conclusion, longer mPFS was observed in patients receiving pazopanib as a second-line therapy for aSTS, rather than in later lines of treatment. Also, mPFS with pazopanib was maintained irrespective of patient age or the use of dose modifications for management of toxicity.

## Additional files

Additional file 1: **Table S1.** Dose modification protocol for potential treatment-related adverse events in the PALETTE trial. This table describes the dose modification protocol used in the PALETTE trial for potential treatment-related adverse events. (DOCX 41 kb)

Additional file 2: Overview of study sites and their affiliated ethics committees. This table presents a list of all study sites/addresses and the affiliated ethics committees. (DOCX 32 kb)

## Data Availability

Novartis is committed to sharing with qualified external researchers access to patient-level data and supporting clinical documents from eligible studies. These requests are reviewed and approved by an independent review panel on the basis of scientific merit. All data provided are anonymized to respect the privacy of patients who have participated in the trial in line with applicable laws and regulations.

## Abbreviations

AE: Adverse event
aSTS: Advanced soft tissue sarcoma
CI: Confidence interval
EMA: European Medicines Agency
GIST: Gastrointestinal stromal tumor
HR: Hazard ratio
IQR: Interquartile range
ITT: Intention to treat
mPFS: Median progression-free survival
OS: Overall survival
PDGF: Platelet-derived growth factor
PDGFR: Platelet-derived growth factor receptor
PFS: Progression-free survival
RCC: Renal cell carcinoma
SAE: Serious adverse event
SD: Standard deviation
STS: Soft tissue sarcoma
TKI: Tyrosine kinase inhibitor
US FDA: United States Food and Drug Administration
VEGF: Vascular endothelial growth factor
VEGFR: Vascular endothelial growth factor receptor
