# Supplementary material for: Safety and efficacy of Pazopanib in advanced soft tissue sarcoma: PALETTE (EORTC 62072) subgroup analyses
Source: BMC Cancer. 2019 Aug 13;19:794. doi: 10.1186/s12885-019-5988-3 (PMC6691522; doi:10.1186/s12885-019-5988-3)
Supplement: Supplementary file 1 — Table S1. Dose modification protocol for potential treatment-related adverse events in the PALETTE trial. This table describes the dose modification protocol used in the PALETTE trial for potential treatment-related adverse events. (DOCX 41 kb) [file 12885_2019_5988_MOESM1_ESM.docx]

**Additional File 1**

| **Table S1. Dose Modification Protocol for Potential Treatment-Related Adverse Events in the PALETTE Trial** | |
| --- | --- |
| **Adverse events and descriptions** | **Dose modification** |
| Hypertension | |
| (A) Asymptomatic and persistent SBP:  ♦ 150 < SBP < 170 mmHg,  ♦ or 90 < DBP < 110 mmHg,  ♦ or a clinically significant increase in DBP of ≥ 20 mmHg | Step 1. Continue study treatment with 800-mg dose  Step 2. Adjust current or initiate new antihypertensive medication(s)  Step 3. Titrate antihypertensive medication(s) during next 2 weeks as indicated to achieve well-controlled BP.^a^ If BP is not well controlled within 2 weeks, follow Step 1 in scenario (B) |
| (B) Symptomatic, or SBP ≥ 170 mmHg,  ♦ or DBP ≥ 110 mmHg,  ♦ or failure to achieve well-controlled BP within 2 weeks in scenario (A) | Step 1. Interrupt study treatment.  Step 2. Adjust current or initiate new antihypertensive medication(s)  Step 3. Titrate antihypertensive medication(s) during next 2 weeks as indicated to achieve well-controlled BP  Step 4. Restart study treatment at lower dose^b^ once BP is well-controlled^a^ |
| (C) Two or more episodes as described above (B) of hypertension despite modification of antihypertensive medication(s) and reduction of study medication dose | Discontinuation of study treatment and follow-up per protocol |
| Proteinuria | |
| UPC < 3 | Continue study treatment with 800-mg/day dose; monitor as clinically indicated |
| UPC ≥ 3 | Step 1: Obtain a 24-hour urine protein  Step 2: If 24-hour urine protein is < 3 g, patient may continue treatment at 800 mg/day  OR  If 24-hour urine protein is ≥ 3 g, interrupt treatment until UPC returns to < 3  Restart therapy at lower dose.^b^ Monitor UPC for the remainder of the overall treatment period. If UPC ≥ 3, obtain a 24-hour urine protein  Step 3: If 24-hour urine protein is ≥ 3 g following repeat dose reductions, discontinue treatment and follow-up per protocol |
| Hemorrhage/Bleeding/Coagulopathy | |
| Grade 1 | Continue study treatment with 800-mg dose; monitor as clinically indicated |
| Grade 2 | Step 1. Interrupt study treatment until the AE resolves to ≤ grade 1  Step 2. Restart treatment with lower dose^b^; monitor as clinically indicated |
| Grade 3 or 4, or recurrent ≥ grade 2 event after dose interruption/reduction | Discontinuation of study treatment and follow-up per protocol  Note: If abnormality is not clearly associated with clinical consequences,  Contact the medical monitors to discuss the potential for continuation of study treatment. If agreed, patient may restart treatment at lower dose^b^ |
| Vascular thrombosis | |
| Grade 2 | Continue study treatment with 800-mg dose; monitor as clinically indicated |
| Grade 3 | Step 1. Interrupt study treatment  Step 2. Start to treat the patient with LMWH  Note: Coumadin is prohibited per protocol  Step 3. Resume study treatment at 800 mg during the period of full-dose anticoagulation if all of the following criteria are met:  ♦ The patient must have been treated with LMWH for at least 1 week  ♦ No grade 3 or 4 hemorrhagic events have occurred while on anticoagulation treatment  ♦ Patient should be monitored as clinically indicated during anticoagulation treatment and after resuming study treatment |
| Grade 4 | Discontinuation of study treatment and follow-up per protocol |
| Thrombocytopenia/Neutropenia | |
| Grade 1 or 2 | Continue study treatment with 800-mg dose; monitor as clinically indicated |
| Grade 3 or 4 | Step 1. Interrupt study treatment until toxicity reduced to ≤ grade 2  Step 2. Restart study treatment with lower dose^b^ |
| Recurrent grade 3/4 event after dose reduction | Discontinuation of study treatment and follow-up per protocol  Note: If patient is benefiting from study treatment, contact the medical monitors to discuss course of action |
| Note: no dose reduction rules are indicated for anemia unless due to hemorrhage or bleeding as noted above | |
| Other clinically significant adverse events | |
| Grade 1 | Continue study treatment with 800-mg dose; monitor as clinically indicated |
| Grade 2 or 3, if clinically  significant | Step 1. Interrupt study treatment until toxicity resolves to ≤ grade 1  Step 2. Restart study treatment at a lower dose^b^; monitor as clinically indicated |
| Recurrent grade 2/3, if clinically  significant | Discontinuation of study treatment and follow-up per protocol |
| Grade 4 | Discontinuation of study treatment and follow-up per protocol  Note: If the patient is benefiting from therapy contact the medical monitors to discuss course of action |
| ^a^Well-controlled BP defined as mean SBP ≤ 150 mmHg and mean DBP ≤ 90 mmHg.  ^b^Dose should be reduced by 200 mg (ie, 800 mg to 600 mg or 600 mg to 400 mg).  BP, blood pressure; DBP, diastolic blood pressure; LMWH, low-molecular-weight heparin; SBP, systolic blood pressure; UPC, urine protein to creatinine ratio. | |
