# Supplementary material for: Safety and efficacy of Pazopanib in advanced soft tissue sarcoma: PALETTE (EORTC 62072) subgroup analyses
Source: BMC Cancer. 2019 Aug 13;19:794. doi: 10.1186/s12885-019-5988-3 (PMC6691522; doi:10.1186/s12885-019-5988-3)
Supplement: Supplementary file 2 — Overview of study sites and their affiliated ethics committees. This table presents a list of all study sites/addresses and the affiliated ethics committees. (DOCX 32 kb) [file 12885_2019_5988_MOESM2_ESM.docx]

**Additional File 2**

**Overview of study sites and their affiliated ethics committees**

| **Investigator no./Center no.** | **Description of Research Facility,**  **Hospital/ Institution, and Address** | **Name of IEC/IRB Committee,**  **Address** |
| --- | --- | --- |
| **Australia** | | |
| 088807/ 063733 | Eastern Clinical Research Unit 16 Arnold Street  Box Hill  VIC, 3128 Australia | Eastern Health Research and Ethics Committee, 5 Arnold Street, Box Hill, VIC, 3128 Australia |
| 115867 / 057300 | Haematology Oncology Clinical Trials, Royal Hobart Hospital, 48 Liverpool Street, Hobart, Tasmania, Australia 7000 | Human Research Ethics Committee (Tasmania) Network, University of Tasmania, Churchill Avenue, Sandy Bay 7005, Hobart, Tasmania, Australia |
| 157501 / 057324 | Ashford Cancer Centre Research, Level 1, Tennyson Centre, 520 South Road, Kurraita Park, SA 5037 Australia | Bellberry Limited, 229 Greenhill Road, Dulwich, South Australia, 5065, Australia |
| 036357/ 057301 | Prince of Wales Hospital, Department of Medical Oncology, Barker street, Randwick NSW 2031, Australia | Cancer Institute NSW Clinical Research Ethics Committee, Cancer Institute NSW, Level 1, Biomedical Building, Australian Technology Park, 1 Central Avenue, Eveleigh NSW 2015, Australia |
| 54418/ 057325 | Sir Charles Gairdner Hospital, Hospital Avenue, Nedlands, WA, Australia 6009 | Sir Charles Gairdner Hospital Human Research Ethics Committee, Sir Charles Gairdner Hospital, Hospital Avenue, Nedlands, 6009, Western Australia, Australia. |
| 158700 / 057670 | Princess Alexandra Hospital, Cancer Services, Ipswich Rd, Woolloongabba, QLD 4102, Australia | The Princess Alexandra Human Research Ethics Committee, Princess Alexandra Hospital, Ipswich Rd, Woolloongabba QLD 4102, Australia |
| **Belgium** | | |
| 092327 / 056945 | University Hospital Gent  Department of Medical Oncology 4B2 De Pintelaan 185  9000 Gent Belgium | Comite d'Ethique lnstitut Jules Bordet  Boulevard de Waterloo 125 1000 Bruxelles  Belgium |
| 093267 / 056943 | Centre Hospitalier Universitaire de Liege  Service d'oncologie medicale Batiment B35  Domaine Universitaire du Sart Tilman 4000 Liege  Belgium | Comite d'Ethique lnstitut Jules Bordet Boulevard de Waterloo 125  1000 Bruxelles Belgium. |
| 054808 / 056941 | Institut Jules Bordet  Department of Medical Oncology Boulevard de Waterloo 125 1000 Brussels  Belgium | Comite d'Ethique lnstitut Jules Bordet  Boulevard de Waterloo 125 1000 BruxellesBelgium |
| 93263/ 056944 | Cliniques Universitaires St-Luc Medical Oncology Unit Avenue Hippocrate 10  1200 Brussels Belgium | Comite d'Ethique Institut Jules Bordet  Boulevard de Waterloo 125 1000 Bruxelles  Belgium |
| 156729 / 056942 | UZ Leuven Gasthuisberg Department of General Medical Oncology  Herestraat 49  3000 Leuven, Belgium | Comité d'Ethique lnstitut Jules Bordet  Boulevard de Waterloo 125 1000 Bruxelles Belgium |
| **Denmark** | | |
| 043354/ 056857 | Herlev Hospital Department of Oncology Herlev Ringyej 75  DK-2730 Herlev, Denmark | De Videnskabsetiske Komiteer for Region Hovedstaden, Regionsgaarden  Kongens Vaenge 2  3400 Hilleroed, Denmark |
| **France** | | |
| 060439 / 057464 | Centre Leon Berard, Department de Medecine, 28 rue laennec, 69373 Lyon  cedex 08- France | Comite de Protection des Personnes Sud-est IV, Centre Leon Berard, 28 rue Laennec, 69373 Lyon cedex 08-France |
| 157848/ 057468 | Institut Bergonie  Medical & Pediatric Oncologie 229 Cours de l'Argonne  33076 Bordeaux cedex - FRANCE | Comite de Protection des Personnes Sud-est IV  Centre Leon Berard 28 rue Laennec  69373 LYON cedex 08 - FRANCE |
| 118927/ 057471 | Insitut de Cancerologie de la Loire Service d'Oncologie  108 bis avenue Albert Raimond BP 60008  42271 St-Priest-en-Jarrez - FRANCE | Comite de Protection des Personnes Sud-est IV  Centre Leon Berard 28 rue Laennec  69373 LYON cedex 08 - FRANCE |
| 078195/ 057466 | C.H.U. De la Timone, Service d'Oncologie Medicale, 264 rue Saint- Pierre, 13385 Marseille - Fance | Comite de Protection des Personnes Sud-est IV, Centre Leon Berard, 28 rue Laennec, 69373 Lyon cedex 08-France |
| 078202/ 057470 | Institut Gustave Roussy Departement d'Oncologie Medical 39 rue Camille Desmoulins  94805 Villejuif - FRANCE | Comite de Protection des Personnes Sud-est IV  Centre Leon Berard 28 rue Laennec  69373 LYON cedex 08 - FRANCE |
| 157852/ 057469 | Centre Oscar Lambret Service d'Oncologie Medicale 3 rue Frederic Combemale BP 307  59020 Lille cedex - FRANCE | Comite de Protection des Personnes Sud-est IV  Centre Leon Berard 28 rue Laennec  69373 LYON cedex 08 - FRANCE |
| 037311/ 057465 | Institut Curie Medecine Oncologique 26 rue d'Ulm  75248 Paris cedex 05 - FRANCE | Comite de Protection des Personnes Sud-est IV  Centre Leon Berard 28 rue Laennec  69373 LYON cedex 08 - FRANCE |
| 050399/ 057467 | CRLCC Alexis Vautrin, Service Medecine Oncologique, Avenue de Bourgogne, 5451 1 Vandoeurnre les Nany - France | Comite de Protection des Personnes Sud-est IV, Centre Leon Berard, 28 rue Laennec, 69373 Lyon cedex 08- France. |
| **Germany** | | |
| 156381/ 057642 | Universitatsklinikum- Essen, Innere Klinik u, Poliklinik, Westdeutsches Tumorzentrum, Hufelandstrasse 55,  45122 Essen, Germany | Ethikkornmission der medizinischen Fakultat der Universitat Koln, Joseph- Stelzmann-Str. 20, 50931 Koln, Germany |
| 156384/ 057643 | Universitatsklinikum Heidelberg, Med. Klinik, lnnere Medizin V, Hamatologie - Onkologie, Im Neuenheimer Feld 410, 69120 Heidelberg, Germany | Ethikkommission der medizinlschen Fakultat der Universitat Koln, Joseph- Stelzmann-Str. 20, 50931 Koln, Germany |
| 133420/ 057644 | Universitatsklinikum Carl Gustav Carus, Medizinische Klinik und Poliklinik I, Onkologische Tagesklinik, Haus  2c, Fetscherstr. 74,01307 Dresden, Germany | Ethikkommission der medizinischen Fakultat der Universitat Koln Joseph-Stelzmann-Str. 20  50931 Koln, Germany |
| 138740/ 057645 | Medizinische Hochschule Hannover, Zentrum Innere Medizin, Hamatologie - Onkologie  Carl-Neuberg-Str, 1, 30625 Hannover, Germany | Ethikkommission der medizinischen Fakultat der Universitat Koln Joseph-Stelzmann-Str. 20  50931 Koln, Germany |
| 156385/ 057646 | Klinikum Mannheim gGmbH, Universitatsklinikum, Chirurgische Klinik, Chirurgische Onkologie, Theodor-Kutzer-Ufer 1-3, 68167 Mannheim, Germany  Deutsches Krebsforschungszentrum, Abt. Onkologische Diagnostik und Therapie, Im Neuenheimer Feld, 280,  69120 Heidelberg, Germany | Ethikkornmission der medizinischen Fakultat der Universitat Koln, Joseph- Stelzmann-Str. 20, 50931 Koln, Germany |
| 156387/ 057647 | Helios Klinikum Bad Saarow, Klinik for Innere Medizin III, Sarkomzentrum, Pieskower Str.33, 15526 Bad Saarow, Germany | Ethikkommission der medizinischen Fakultat der Universitat Koln, Joseph- Stelzmann-Str. 20, 50931 Koln, Germany |
| 158542/ 057652 | Klinikum der J.W. Goethe Universitat, Zentrum der Inneren Medizin, Med Klinik II - Hamatologie/Onkologie, Theodor-Stern-Kai 7, 60590 Frankfurt, Germany | Ethikkommission der medizinischen Fakultat der Universitat Koln Joseph-Stelzmann-Str. 20  50931 Koln, Germany |
| 156404/ 057641 | Klinikum der Universitat zu Koln, Klinik I Fuer Innere Medizin, Kerpener Str.  62, 50924 Koln, Germany | Ethikkommission der medizinischen Fakultat der Universitat Koln, Joseph- Stelzmann-Str. 20, 50931 Koln, Germany |
| **Italy** | | |
| 053156/ 057264 | Divisione Di Dncologia Medica-I.Rc,C.  -Ad ordine Mauriziano Di Torino Strada Provinciale 142 -10060 Candiolo (To) Italy | Coordinating Ethical Committee: COMITATO ETICO INDIPENDENTE FONDAZIONE IRCCS "ISTITUTO NAZIONALE DEI TUMORI"  VIA VENEZIAN 1  20133 MILANO ITALY  Local Ethical Comittee: COMITATO ETICO DELLE AA.OO O.I.R.M\S.ANNA  ORDINE MAURIZIANO DI  TORINO,CORSO SPEZIA 60 - 10126 TORINO, ITALY |
| 158434/ 057632 | SSD Oncologia Medica - Sarcomi Ossa e Tessuti Molli - 1st. Naz. Studio e Cura Tumori - Fond. Pascale -Via M. Semmola, 3 - 80131 Napoli- ltaly | Coordinating Ethical Committee: Comitato Etico Indipendente - Fondazione Irccs " Istituto Nazionale Dei Tumori" -Via Venezian 1 - 20133 Milano – Italy  Local Ethical Committee: Comitato Etico lndipendente - lstiuto Nazionale Studio e Cura Tumori - Fondazione Pascale -Via Mariano Semola, 3 - 80131 Napoli - ltaly |
| 156710/ 057017 | Struttura semplice Trattamendo Medico Sarcomi dell'Adulto - Departimento Medicina Oncologica Fondazione IRCCS" Istituto Nazionale dei Tumori'" Via Venezian 1, 20133  Milano, Italy | Comitato Etico Indipendente Fondazione Irccs" Istituto Nazionale Dei Tumori"  Via Venezian 1  20133 Milano,Italy |
| 053155/ 057266 | UOA ONCOLOGIA  OSPEDALE" GRADENIGO" - C.so  Regina Margherita 8,10153 Torino, Italy | Coordinating Ethical Committee: COMITATO ETICO INDIPENDENTE FONDAZIONE IRCCS "ISTITUTO NAZIONALE DEI TUMORI"  VIA VENEZIAN 1  20133 MILANO ITALY  Local Ethical Comittee REGIONE PIEMONTE  COMITATO ETICO DELL'ASL TO 2 CORSO REGINA MARGHERITA  153bis  10152 TORINO, ITALY. |
| 156711/ 057018 | Istituto Nazionale Tumori Regina Elena Divisione di Oncologia Medica A Via E. Chianesi, 53  00144 Roma Italy | Coordinating Ethical Committee: COMITATO ETICO INDIPENDENTE FONDAZIONE IRCCS "ISTITUTO NAZIONALE DEI TUMORI"  VIA VENEZIAN 1  20133 MILANO ITALY.  Local Ethical Committee: Comitato Etico IF.O. Istituto Fisioterapici Ospitalieri IRCCS Regina Elena - IRCCS San  Gallicano, Via E. Chianesi, 53 - 00144 Roma, Italy |
| 013219/ 057019 | Struttura Complessa di Oncologia Azienda Ospedaliera Santa Maria Via Tristano di Jaonnuccio, 1 05100 Terni - Italy | Coordinating Ethical Committee: COMITATO ETICO INDIPENDENTE FONDAZIONE IRCCS "ISTITUTO NAZIONALE DEI TUMORI"  VIA VENEZIAN 1  20133 MILANO ITALY.  Local Ethical Committee: Comitato Etico Azleodie Sanitarie Umbria (CEAS)  Segreteria Scientlfica Amministrativa via DeIta RivoluZione, 16  0670 EIera di Corciano (PG) |
| 085014/ 057267 | Oncologia Medica Ed Ematologia Istituto Clinico Humanitas  Via Manzoni 56, 20089 Rozzano, (Mi), Italy | Coordinating Ethical Committee: COMITATO ETICO INDIPENDENTE FONDAZIONE IRCCS "ISTITUTO NAZIONALE DEI TUMORI"  VIA VENEZIAN 1  20133 MILANO ITALY  Local Ethical Committee:  COMITATO ETICO ASL MILANO DUE AZIENDA SANITARIA LOCALE DELLA PROVINCIA MILANO 2  VIA VIII GIUGNO, 69  20077 MELEGNANO (MI)-ITALY. |
| 069052/ 057265 | Divisione Di Oncologia Medica Falck Ospedale Niguarda Ca' Granda Piazza Ospedale Maggiore 3  20162 Milano, Italy | Coordinating Ethical Committee: COMITATO ETICO INDIPENDENTE FONDAZIONE IRCCS "ISTITUTO NAZIONALE DEI TUMORI"  VIA VENEZIAN 1  20133 MILANO ITALY  Local Ethical Comittee COMMISSIONE ETICO.SC1ENTIFICA DELL'OSPEDALE  OSPEDALE CA' GRANDA NIGUARDA PlAZZA OSPEDAlE MAGGIORE 3  20162 MILANO ITALY |
| **Japan** | | |
| 152506 / 055522 | Department of Orthopaedic surgery, Osaka Medical Center for Cancer and Cardiovascular Diseases,  1-3-3, Nakamichi, Higashinari-ku,  Osaka-shi, Osaka, 537-851 1, Japan | Institutional Review Board.Osaka Medical Center for Cancer and Cardiovascular Diseases,  1-3-3, Nakamichi, Higashinari-ku,  Osaka-shi, Osaka, 537-851 1, Japan |
| 152500 / 055518 | National Cancer Center Hospital  5-1-1, Tsukiji, Chuo-ku, Tokyo, 104-  0045, Japan | National Cancer Center Hospital  5-1 -1, Tsukiji, Chuo-ku, Tokyo, 104-  0045, Japan |
| 152493 / 055517 | Chiba Cancer Center  Department of Orthopaedic surgery, Chiba cancer centre,  666-2, Nitona-cho, Chuo-ku. Chiba-shi, Chiba, 260-8717, Japan | Institutional Review Board, Chiba Cancer Center  666-2, Nitona-cho, Chuo-ku, Chiba-shi, Chiba, 260-8717, Japan |
| 152510 / 055524 | National Hospital Organization Hokkaido Cancer Center  2-3-54, Kikusui 4-jo, Shiroishi- ku,,Sapporo-shi, Hokkaido, 003-0804, Japan | National Hospital Organization Hokkaido Cancer Center  2-3-54, Kikusui 4-jo, Shiroishi-ku, Sapporo-shi, Hokkaido, 003-0804, Japan |
| 152509 / 055523 | Mie University Hospital  2-174, Edobashi, Tsu-shi, Mie, 514-  8507, Japan | The Institutional Review Board of Mie University Hospital  2-174, Edobashi, Tsu-shi, Mie, 514-  8507, Japan |
| 152501 / 055519 | Okayama University Hospital  2-5-1, Shikata-cho,Kita-Ku, Okayama- city, Okayama, 700-8558, Japan | The Institutional Review Board of Okayama University Hospital  2-5-1, Shikata-cho, Okayama-city,  Okayama, 700-8558, Japan |
| 152492 / 055516 | Department of Orthopaedic surgery, Aichi cancer centre Hospital,  1-1, Kanokoden, Chikusa-ku, Nagoya- shi, Aichi, 464-8681, Japan | Institutional Review Board, Aichi Cancer Center Hospital  1-1, Kanokoden, Chikusa-ku, Nagoya- shi, Aichi, 464-8681, Japan |
| 152504 / 055521 | Department of Orthopaedic surgery, National Hospital Organization Osaka National Hospital,  2-1-14, Hoenzaka, Chuo-ku, Osaka- shi, Osaka, 540-0006, Japan | Institutional Review Board,National Hospital Organization Osaka National Hospital,  2-1-14, Hoenzaka, Chuo-ku, Osaka- shi, Osaka, 540-0006, Japan |
| 152503 / 055520 | Division of Orthopaedic Surgery, National Hospital Organization Kyushu Cancer Center  3-1-1, Notame, Minami-ku, Fukuoka- shi, Fukuoka, 81 1-1395, Japan | Institutional Review Board.National Hospital Organization Kyushu Cancer Center  3-1-1, Notame, Minami-ku, Fukuoka- shi, Fukuoka, 81 1-1395, Japan |
| **Korea** | | |
| 114907 / 057299 | Asan Medical Center, 388-1, Pungnap- dong, Songpa-gu, Seoul, 138-736, Korea | Asan Medical Center, 388-1, Pungnap- dong, Songpa-gu, Seoul, 138-736, Korea |
| 161249/ 057388 | Seoul National University Hospital, 28 Yongon-dong, Chongno-gu, Seoul, Korea, 110-744 | Seoul National University Hospital, 28 Yongon-dong, Chongno-gu, Seoul, Korea, 110-744 |
| 146081/ 067754 | Samsung Medical Center, 50 Irwon- dong, Gangnam-gu, Seoul 135-710, KOREA | Samsung Medical Center, 50 Irwon- dong, Gangnam-gu, Seoul 135-710, Korea |
| 157500 / 057391 | Youngnam University Hospital, 317-1, Daemyung-Dong, Nam-gu, Daegu, 705-717, Korea | Youngnam University Hospital, 317-1, Daemyung-Dong, Nam-gu, Daegu, 705-717, Korea |
| 093248 / 057298 | Severance Hospital, YUCM,134 Shinchon-dong, Seodaemun-gu, Seoul, Korea, 120-752 | Severance Hospital, YUCM,134 Shinchon-dong, Seodaemun-gu, Seoul, Korea, 120-752 |
| 157487 / 057390 | National Cancer Center, 809, Madu- dong, Ilsan-gu, Goyang-si, Gyeonggi- do, 410-769, Korea | National Cancer Center, 809, Madu- dong, Ilsan-gu, Goyang-si, Gyeonggi- do, 410-769, Korea |
| **Netherlands** | | |
| 078196 / 055123 | LUMC  Dept.Oncology Albinusdreef 2  2333 ZA Leiden, The Netherlands | Prinsengracht 83 1015 DN Amsterdam  The Netherlands |
| 149000 / 055126 | University Medical Center Groningen Div. of Medical Oncology Hanzeplein 1  9713 GZ Groningen  The Netherlands | Prinsengracht 83 1015 DN Amsterdam  The Netherlands |
| 078210 / 055122 | University Medical Center Nijmegen- Radboud (UMCN)  Department of Medical Oncology Geert Grooteplein 10  6525 GA Nijmegen  The Netherlands | IRB  Prinsengracht 83  1015 DN Amsterdam  The Netherlands |
| 001766 / 055124 | The Netherlands Cancer Institute- Antoni Van Leeuwenhoekziekenhuis (NKI-AvL)  Department of Medical Oncology Plesmanlaan 121  1066 CX Amsterdam  The Netherlands | IRB  Prinsengracht 83  1015 DN Amsterdam  The Netherlands |
| 128817/ 055125 | Erasmus MC  location Daniel, Medical Oncology Groene Hilledijk 301  3075 EA Rotterdam  The Netherlands | IRB  Prinsengracht 83  1015 DN Amsterdam  The Netherlands |
| **Spain** | | |
| 099108/ 057676 | Hospital Clinico San Carlos C/Dr. Martin Lagos, s/n 28040 Madrid-Spain | Reference Ethics Committee: Comite Etico de Investigacion Clinca Hospital Clinico San Carlos, Dr, Marlin Lagos s/n, 28040 Madrid, Spain |
| 158732/ 057699 | Hospital 12 de Octubre, Avda. de Cordoba,s/n, 28041 Madrid-SPAIN | Reference Ethics Committee: Comite Etico de Investigacion Clinica Hospital Clinico San Carlos, Dr. Martin Lagos, s/n, 28040 Madrid, Spain. |
| 158815/ 057700 | Hospital Universitari Son Espases Servicio de Oncologia Medica Ctra. de VaUdemossa, 79  07010 Palma de Mallorca SPAIN | Reference Ethics Committee: Comite Etico de Invesligaci6n Cllnica Hospital Clinico San Carlos, Or. Martin Lagos sin, 28040 Madrid, Spain  Local Ethics Committee: Comite ttie d'lnvestigaci6 Cllnica Illes Balears, Conselleria de Salut i Consum, Ceeili Metel,  18,07003 Palma de Mallorca, Spain |
| 100298/ 057677 | Instituto Valenciano de Oncologia, Servicio de Oncologia Medica, Profesor Beltran Baguena 11, 8, 19.  46009 Valencia- Spain | Reference Ethics Committee: Comite Etico de Investigacion Clinica Hospital Clinico San Carlos, Dr. Martin Lagos, s/n, 28040 Madrid, Spain.  Comite Etico de Investigacion Cllnica- Instituto Valenciano de Oncologia- C/Profesor Beltran Baguena, 8- 46009 VALENCIA-Spain |
| Sweden | | |
| 155578 / 056544 | Onkologiska Kliniken Akademiska Sjukhuset SE-751 85 UPPSALA, Sweden | Regionala Etikpovningsnamnden I Lund Stora Algatan 4 SE-223 50 Lund, Sweden |
| 155552 / 056542 | SU/Sahlgrenska Universitetssjukhuset Onkologiska enheten Jubileumskliniken  SE- 413 45 GOTEBORG, Sweden | Regionala Etikprovningsnamnden i Lund  Stora Algatan 4  SE-223 50 LUND, Sweden |
| 155550 / 056540 | Universitetssjukhuset Lund Onkologkliniken SE-221 85 Lund, Sweden | Regionala Etikprovningsnamnden I Lund  Stora Algatan 4  SE-223 50 LUND, Sweden |
| 155550 / 056541 | Onkologiska Kliniken Norrlands Universitetssjukhus SE-901 85 Umea,Sweden | Regionala Etikpovningsnamnden I Lund Stora Algatan 4  SE-22350 LUND, Sweden |
| 155553 / 056543 | Onkologiska Kliniken Universitetssjukhuset  SE-581 85 LINKOPING, Sweden | Regionala Etikprovningsnamnden i Lund  Stora Algatan 4  SE-223 50 LUND, Sweden |
| **UK** | | |
| 145876/ 062882 | Nottingham University Hospitals NHS Trust Hucknall Road Nollingham,United Kingdom NG51PB | North West Research Ethics Committee, NHS North West, Room 155 - Gateway House, Piccadilly South,  Manchester. United Kingdom, M60 7LP.  Nottingham Research Ethics Commrttee, Room 203, Level 2, 1 Standard Court, Park Row, Nottingham. United  KingdDm, NG1 6GN. |
| 078199/ 057927 | The Royal Marsden Hospital, 203 Fulham Road, London, SW3 6JJ, United Kingdom | North West Research Ethics Committee, NHS North West, Room 155 - Gateway House, Piccadilly South, Manchester, United Kingdom, M60 7LP  The Royal Marsden Hospital Research Ethics Committee St Georges University of London, South London REC  Office 1, 1st Floor - Jenner Wing, Blackshaw Road, Tooting, London SW17 ORE |
| 078203/ 057928 | Christie Hospital, Wilmslow Road, Manchester, M20 4BX, United Kingdom | North West Research Ethics Committee, NHS North West, Room 155 - Gateway House, Piccadilly South,  Manchester, United Kingdom, M60 7LP  South Manchester Research Ethics Committee, Great Manchester  Strategic Health Authority, Room 181, 1St Floor,  Gateway House, Piccadilly South, Manchester, M60 7LP, United Kingdom. |
| 159208/ 057929 | Leeds Teaching Hospitals NHS Trust, Institute of Oncology, St James's University Hospital, Beckett Street, Leeds,  LS97TF | North West Research Ethics Committee, NHS North West, Room 155 - Gateway House, Piccadilly South,  Manchester, M60 7LP United Kingdom  Leeds (East) Research Ethics Committee, Clinical Sciences Building, St James's University Hospital, Becket Street,  Leeds LS9 7TF United Kingdom |
| 159226/ 057932 | Beatson West of Scotland Cancer Centre, 1053 Great Western Road, Glasgow, United Kingdom, G12 OYH | North West Research Ethics Committee, NHS North West, Room 155 - Gateway House, Piccadilly South, Manchester. United Kingdom, M60 7LP.  West Glasgow Ethics Committee, Western Infirmary, Dumbarton Road, Glasgow. United Kingdom, GI1 6NT |
| 078212/ 057933 | Weston Park Hospital, Whitham Road, Sheffield. UK, S10 2SJ | North West Research Ethics Committee, 155, Gateway House, Piccadilly South, Manchester. UK.M60 7PL  North Sheffield Research Ethics Committee, Northern General Hospital, Herries Road. Sheffield. UK S5 7AU. |
| **USA** | | |
| 010638 / 056518 | Sarcoma Oncology Center 2811 Wilshire Blvd  Suite 414  Santa Monica, CA 90403 | Western Institutional Review Board 3535 7th Avenue, SW  Olympia, WA 98502 |
| 148892/ 056524 | UAB, Comprehensive Cancer Centre, The Kirklin Clinic at Acton Road, 2145 Bonner Way, Birmingham, AL 35243 | Westen Institutional Review Board, 3535 Seventh Avenue SW, Olympia, WA 98502-5010 |
| 079760/ 056523 | University Hospitals Case Medical Center, 11100 Euclid Avenue, Cleveland, United States, OH 44106 | Case Western Reserve University School of Medicine, Office of Case Cancer Institutional Review Board, 10900 Euclid Ave. Cleveland, OH 44106 |
| 031108 / 056525 | Dana- Farber Cancer Institute (DFCI), 44 Binney St, Boston, MA 02115, USA | Dana-Farber Cancer Institute Institutional Review Board, 44 Binney Street, Boston, MA 02115, OS-200 |
| 031108 / 065165 | Massachusetts General Hospital, 55 Fruit Street, Boston, MA 02114 | Dana-Farber Cancer Institute Institutional Review Board, 44 Binney Street, Boston, MA 02115, OS-200 |
| 155695/ 056553 | Cedars-Sinai Medical Ctr., Samuel Oschin Cancer Ctr., 8700 Beverly Blvd., Room AC1237, Los Angeles, Ca 90048 | Cedars-Sinai Medical Center, Institutional Review Board, 8383 Wilshire Blvd, Suite 742, Beverly Hills, CA 90211 |
| 031146/ 056531 | University of California, lrvine Medical Center, Chao Family Comprehensive Cancer Center, 101 The City Drive, Bldg. 23, Orange, CA. 92868 | University of California-Irvine, Institutional Review Board Office of Research Administration, 300 University Tower, lrvine, CA. 92697- 7600 |
| 120725 / 057819 | Advocate illinois Masonic Medical Center, Creticos Cancer Center, 901  W. Wellington Ave., Chicago, Illinois 60657 | Advocate Health Care Institutional Review Board, 205 W. Touhy Ave., Suite 203, Park Ridge, Illinois 60068 |
| 155696 / 056554 | University of Minnesota, Medical Center, Fairview, 420 Delaware Street SE, Minnoapolis, MN 55455 | Research Subjects, Protection Programs, University of Minnesota, D- 528 Mayo Memorial Building, MMC 820, 420 Delaware Street SE,  Minneapolis, MN 55455 |
| 089161 / 056533 | Pennsylvania Oncology Hematology Associates  230 West Washington Square, 2nd Floor  Philadelphia, PA 19106 | Institutional Review Board Pennsylvania Hospital 800 Spruce Street  Philadelphia, PA 19107 |
